# Increased sagittal abdominal diameter is associated with a higher risk of kidney stones

**DOI:** 10.1371/journal.pone.0317717

**Published:** 2025-01-24

**Authors:** Wei Song, Shugen Li, Guangchun Wang, Shang Gao

**Affiliations:** 1 Department of Urology, Shanghai Tenth People’s Hospital, School of Medicine, Tongji University, Shanghai, China; 2 Department of Urology, Suzhou Wuzhong No.2 People’s Hospital, Suzhou, China; Sichuan University, CHINA

## Abstract

**Background:**

This study investigates the relationship between sagittal abdominal diameter (SAD), a measure of abdominal obesity, and kidney stone disease (KSD) in the U.S. population. Additionally, it explores potential underlying mechanisms and evaluates the clinical utility of a predictive model.

**Methods:**

Data were collected from 11,671 participants, including 1,136 cases of KSD. Univariate and multivariate logistic regression analyses, dose-response curves, and mediation effect assessments were employed to examine the association between SAD and KSD. A predictive model was developed and validated using calibration curves, receiver operating characteristic (ROC) curves, and clinical decision curves. Additionally, hematological indicators were analyzed to identify potential mediating factors.

**Results:**

SAD showed a strong and positive association with KSD, even after adjusting for confounders such as gender, age, and education. The predictive model demonstrated moderate accuracy (AUC =  0.661) and clinical utility. Hematological analyses indicated that granulocyte count (GRAN) significantly mediated the relationship between SAD and KSD (P <  0.001).

**Conclusions:**

SAD is a significant risk factor for KSD, underscoring the role of abdominal obesity in kidney stone formation. The predictive model demonstrates potential clinical applications for early risk assessment and management of KSD.

## Introduction

Kidney stone diseases (KSD) arises from the abnormal accumulation of crystalline materials (e.g., calcium, oxalic acid, uric acid, cystine) in the kidneys and is a prevalent condition of the urinary system [1,2]. The global prevalence of KSD is high, affecting approximately 10% of adults, with its incidence steadily increasing. This trend poses a significant healthcare challenge in both developed and developing countries, exerting a substantial socio-economic burden and straining healthcare systems [3,4]. Despite the availability of effective treatment modalities, KSD is characterized by a high recurrence rate following stone removal [5]. Consequently, studying the risk factors for kidney stones and implementing preventive strategies for both initial and recurrent stones are essential to reducing the burden on patients. The etiology of KSD is multifactorial, with contributing factors including obesity, diabetes, hypertension, and metabolic disorders [6].

Changes in dietary habits and socio-economic development have elevated abdominal obesity into a serious global health concern [7]. Studies have demonstrated that abdominal obesity increases the risk of kidney stones, potentially through mechanisms such as insulin resistance, and a positive correlation between the two has been observed [8,9]. Traditional measures of abdominal obesity, such as body mass index (BMI) and waist circumference (WC), have been widely used in previous studies [10,11]. However, these indicators do not accurately capture abdominal obesity, as aging can lead to weight loss accompanied by increased abdominal fat [12]. To address this limitation, sagittal abdominal diameter (SAD) has emerged as a novel metric for assessing abdominal and visceral obesity. SAD measures the thickness between the back and the upper abdomen, offering a more precise evaluation of abdominal fat distribution [13]. However, the relationship between SAD and KSD remains unclear, and its utility as a predictor of KSD has yet to be established.

To address this gap, our study utilized data from the National Health and Nutrition Examination Survey (NHANES) spanning 2011–2016, providing a comprehensive and up-to-date dataset to investigate the correlation between SAD and KSD. This research represents a significant advancement by introducing SAD as a novel indicator of obesity-related risk, offering an alternative to traditional BMI-based approaches. The findings provide valuable insights into the role of visceral fat in KSD, with implications for improving risk stratification and enhancing understanding of the metabolic mechanisms underlying KSD development.

## Materials and methods

### 2.1 Study population

NHANES conducts a stratified, multistage probability survey of non-institutionalized U.S. residents. For this study, we analyzed cross-sectional data from three NHANES cycles (2011–2012, 2013–2014, and 2015–2016). Additional details about the data can be accessed on the NHANES website (www.cdc.gov/nchs/NHANES/). The authors are responsible for ensuring the accuracy and integrity of the work and for addressing any related questions. This study was conducted in accordance with the Declaration of Helsinki (as revised in 2013). The Ethics Committee of Suzhou Wuzhong No.2 People’s Hospital approved the study submission and waived the need for ethical approval, as the data used were obtained from public databases.

**Fig 1:**
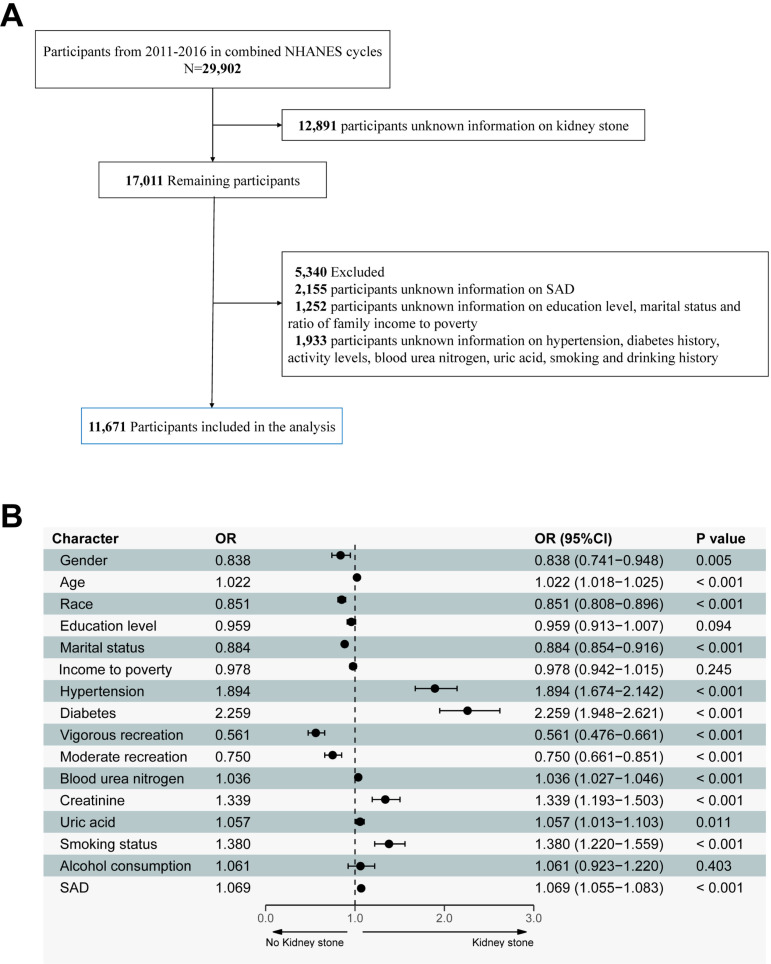
**A.** Study population description for analysis. **B.** Univariate logistic regression results for gender, age, race, education level, marital status, income to poverty, hypertension, diabetes, vigorous recreational activity, moderate recreational activity, blood urea nitrogen, creatinine, uric acid, smoking status, alcohol consumption and SAD for all participants.

From 2011 to 2016, NHANES included 29,902 participants. We excluded individuals based on the following criteria: (I) those without data on KSD (n =  12,891); (II) those without SAD measurements (n =  2,155); (III) those lacking data on education level, marital status, and the ratio of family income to poverty (n =  1,252); and (IV) those missing information on hypertension, diabetes history, activity levels, blood urea nitrogen, uric acid, smoking, and drinking history (n =  1,933). After applying these criteria, 11,671 participants were included in the final analysis (Fig 1A).

### 2.2 Covariate

The covariates in this study included gender (male or female), age, race (Mexican American, Other Hispanic, Non-Hispanic white, Non-Hispanic black, Other race), education (Less than 9th grade, 9–11th grade (Includes 12th grade with no diploma), High school graduate/GED or equivalent, Some college or AA degree, College graduate or above), marital status (Married, Widowed, Divorced, Separated, Never married, Living with partner), household poverty rate, hypertension, diabetes, blood urea nitrogen, creatinine, uric acid, alcohol consumption, smoking, vigorous recreational activity, moderate recreational activity, SAD, and KSD. Hypertension, diabetes, and KSD were defined as self-reported diagnoses.

### 2.3 Definition of SAD

The primary aim of this study was to investigate the effect of SAD on KSD and assess its potential as a clinical predictor of SKD. SAD was measured with participants in a supine position, knees bent at a 90-degree angle, hands placed on the chest, and feet flat on a tabletop. The NHANES examiner located the uppermost margins of the right and left iliac bones and marks the abdomen along the horizontal line of the iliac bones. The distance (in centimeters) between the lower abdomen and the abdominal mark was measured using abdominal calipers, with an accuracy of 0.1 cm. SAD measurements were applicable to subjects aged 8 years and older, excluding pregnant women. In this study, a threshold SAD of 22.4 cm was defined based on dose-response curve results.

### 2.4 Assessment of KSD

The outcome variable, KSD, was determined using NHANES. Participants were categorized into KSD and non-KSD groups based on their response to the standardized question: “Have you ever had a kidney stone?”.

### 2.5 Data analysis

NHANES uses weighted sampling to account for the study design. Continuous variables were described using mean ±  standard deviation (SD), while categorical variables were reported as proportions. Chi-square tests were used to evaluate the clinical characteristics of participants. Univariate and multivariate logistic regression models were employed to calculate adjusted odds ratios (ORs) and 95% confidence intervals (CIs) for the risk of KSD. A logistic regression model was developed to analyze the association between SAD and KSD. The data were split into a training set (50%) and a validation set (50%). Model performance was assessed using calibration curves, discrimination curves, and clinical decision curves. Additionally, mediation analysis was conducted to explore whether the relationship between SAD and KSD was mediated by granulocyte count (GRAN). Using the R package mediation, the total effect (TE) of SAD on KSD was decomposed into direct effects (DE) and indirect effects (IE), with the proportion of IE to TE reflecting the mediation efficacy [14,15]. This approach hypothesized that the association between SAD (X) and KSD (Y) was influenced by an intermediate factor (M). All statistical analyses were performed using R software (version 4.1.3) and SPSS software (version 24.0), with statistical significance set at P <  0.05 (two-sided).

## Results

### Participant characteristics

A total of 11,671 participants meeting the NHANES database criteria from 2011 and 2016 were included in this study. Table 1 shows the baseline clinical characteristics of all participants, which were similar across the cohort. Clinical characteristics, including gender, age, race, education level, marital status, ratio of family income to poverty, hypertension, diabetes, blood urea nitrogen, blood creatinine, blood uric acid, alcohol consumption, smoking, vigorous physical activity, moderate physical activity, and SAD, were assessed using chi-square tests (P <  0.001).

**Table 1:** Baseline characteristics of NHANES participants between 2011–2016 (n = 11671)^a^.

Characteristic	All	None Kidney Stone	Kidney Stone	P value
	patients	90.3 (%)	9.7 (%)	
	N = 11671	N = 10535	N = 1136	
Gender (%)				**0.005**
Male	5895 (50.5)	5276 (50.1)	619 (54.5)	
Female	5776 (49.5)	5259 (49.9)	517 (45.5)	
Age	48.00 [34.00, 63.00]	47.00 [33.00, 62.00]	55.00 [42.00, 67.00]	**<0.001**
Race (%)				** <0.001**
Mexican American	1554 (13.3)	1394 (13.2)	160 (14.1)	
Other Hispanic	1236 (10.6)	1101 (10.5)	135 (11.9)	
Non-Hispanic white	4736 (40.6)	4149 (39.4)	587 (51.7)	
Non-Hispanic black	2467 (21.1)	2322 (22.0)	145 (12.8)	
Other race	1678 (14.4)	1569 (14.9)	109 (9.6)	
Education level (%)				0.05
Less than 9th grade	956 (8.2)	855 (8.1)	101 (8.9)	
9–11th grade (Includes 12th grade with no diploma)	1405 (12.0)	1259 (12.0)	146 (12.9)	
High school graduate/GED or equivalent	2567 (22.0)	2329 (22.1)	238 (21.0)	
Some college or AA degree	3668 (31.4)	3281 (31.1)	387 (34.1)	
College graduate or above	3075 (26.3)	2811 (26.7)	264 (23.2)	
Marital status (%)				**<0.001**
Married	5928 (50.8)	5274 (50.1)	654 (57.6)	
Widowed	775 (6.6)	690 (6.5)	85 (7.5)	
Divorced	1294 (11.1)	1137 (10.8)	157 (13.8)	
Separated	384 (3.3)	346 (3.3)	38 (3.3)	
Never married	2336 (20.0)	2213 (21.0)	123 (10.8)	
Living with partner	954 (8.2)	875 (8.3)	79 (7.0)	
Ratio of family income to poverty	2.51 (1.64)	2.51 (1.64)	2.46 (1.61)	0.245
Hypertension (%)				**<0.001**
Yes	4162 (35.7)	3599 (34.2)	563 (49.6)	
No	7545 (64.3)	6936 (65.8)	573 (50.4)	
Diabetes (%)				**<0.001**
Yes	1554 (13.3)	1283 (12.2)	271 (23.9)	
No	10117 (86.7)	9331 (97.8)	730(71.7)	
Blood urea nitrogen, mg/dL	13.00 [10.00, 16.00]	13.00 [10.00, 16.00]	14.00 [11.00, 17.00]	**<0.001**
Blood creatinine, mg/dL	0.85 [0.72, 1.01]	0.85 [0.71, 1.00]	0.90 [0.74, 1.05]	**<0.001**
Blood uric acid, mg/dL	5.30 [4.40, 6.30]	5.30 [4.40, 6.30]	5.50 [4.50, 6.50]	**0.018**
Alcohol consumption (%)				0.423
Yes	8518 (73.0)	7677 (72.9)	841 (74.0)	
No	3153 (27.0)	2858 (27.1)	295 (26.0)	
Smoking (%)				**<0.001**
Yes	5096 (43.7)	4518 (42.9)	578 (50.9)	
No	6575 (56.3)	6017 (57.1)	558 (49.1)	
Vigorous recreational activity (%)				**<0.001**
Yes	2884 (24.7)	2700 (25.6)	184 (16.2)	
No	8787 (75.3)	7835 (74.4)	952 (83.8)	
Moderate recreational activity (%)				**<0.001**
Yes	5087 (43.6)	4663 (44.3)	424 (37.3)	
No	6584 (56.4)	5872 (55.7)	712 (62.7)	
SAD	22.40 [19.50, 25.70]	22.30 [19.40, 25.50]	23.80 [20.98, 27.00]	**<0.001**
Abdominal Obesity				**<0.001**
Yes	5903 (50.6)	5184 (49.2)	719 (63.3)	
No	5768 (49.4)	5351 (50.8)	417 (36.7)	

^a^For categorical variables, P values were analyzed by chi-square tests. For continuous variables, the t-test for slope was used in generalized linear models.

### SAD and KSD

**Fig 2:**
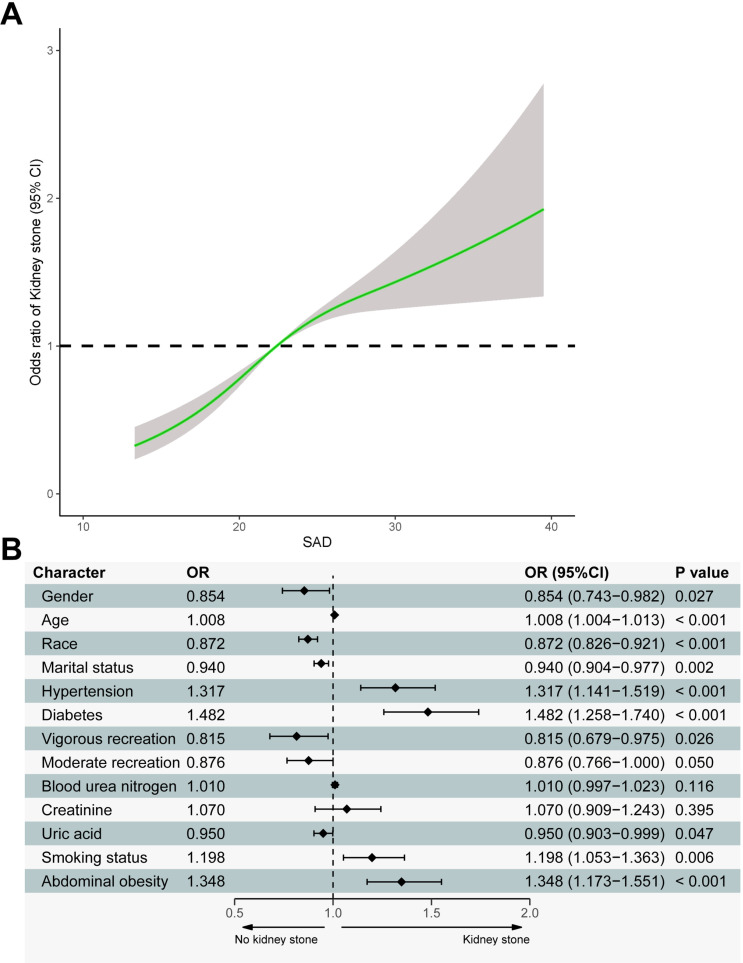
**A.** Dose-response relationship between SAD and KSD. **B.** Results of multivariate logistic regression analysis of gender, age, race, marital status, hypertension, diabetes, vigorous recreational activity, moderate recreational activity, blood urea nitrogen, creatinine, uric acid, smoking status and abdominal obesity with prevalence of KSD for all participants.

Univariate logistic regression analysis was used to evaluate the relationship between gender, age, race, education level, marital status, ratio of family income to poverty, hypertension, diabetes, blood urea nitrogen, creatinine, uric acid, alcohol consumption, smoking, vigorous recreational activity, moderate recreational activity, SAD, and KSD among the 11,671 participants in Fig 1B. Significant associations were observed for the following variables: (OR: 0.838, 95% CI: 0.741–0.948), age (OR:1.022, 95% CI: 1.018–1.025), race (OR:0.851, 95% CI: 0.808–0.896), marital status (OR:0.884, 95% CI: 0.854–0.916), hypertension (OR:1.894, 95% CI: 1.674–2.143), diabetes (OR:2.259, 95% CI: 1.948–2.621), vigorous recreation activity (OR:0.561, 95% CI:0.476–0.661), moderate recreation activity (OR: 0.750, 95% CI:0.661–0.851), blood urea nitrogen (OR:1.036, 95% CI:1.027–1.046), blood creatinine (OR: 1.339, 95% CI:1.193–1.503), blood uric acid (OR: 1.057,95%CI: 1.013-1.103), smoking status (OR:1.380,95%CI:1.220–1.559) and SAD (OR:1.069, 95% CI:1.055–1.083). Dose-response analysis using a restricted cubic bar chart indicated a positive association between SAD and KSD. From this analysis, an SAD threshold of 22.4 cm for abdominal obesity was determined (Fig 2A). Multivariate logistic regression analysis identified statistically significant predictors of KSD (Fig 2B): gender (OR:0.854, 95%CI:0.743–0.982), age (OR:1.008, 95% CI:1.004–1.013), race (OR:0.872, 95% CI:0.826–0.921), marital status (OR:0.940, 95%CI:0.904–0.977), hypertension (OR:1.317, 95% CI:1.141–1.519), diabetes (OR:1.482, 95%CI:1.258–1.740), vigorous recreation activity (OR:0.815, 95%CI:0.679–0.975), uric acid (OR:0.950, 95%CI:0.903–0.999), smoking status (OR:1.198, 95%CI:1.053–1.363) and abdominal obesity (OR:1.348, 95%CI:1.173–1.551).

### Model development and validation

**Fig 3:**
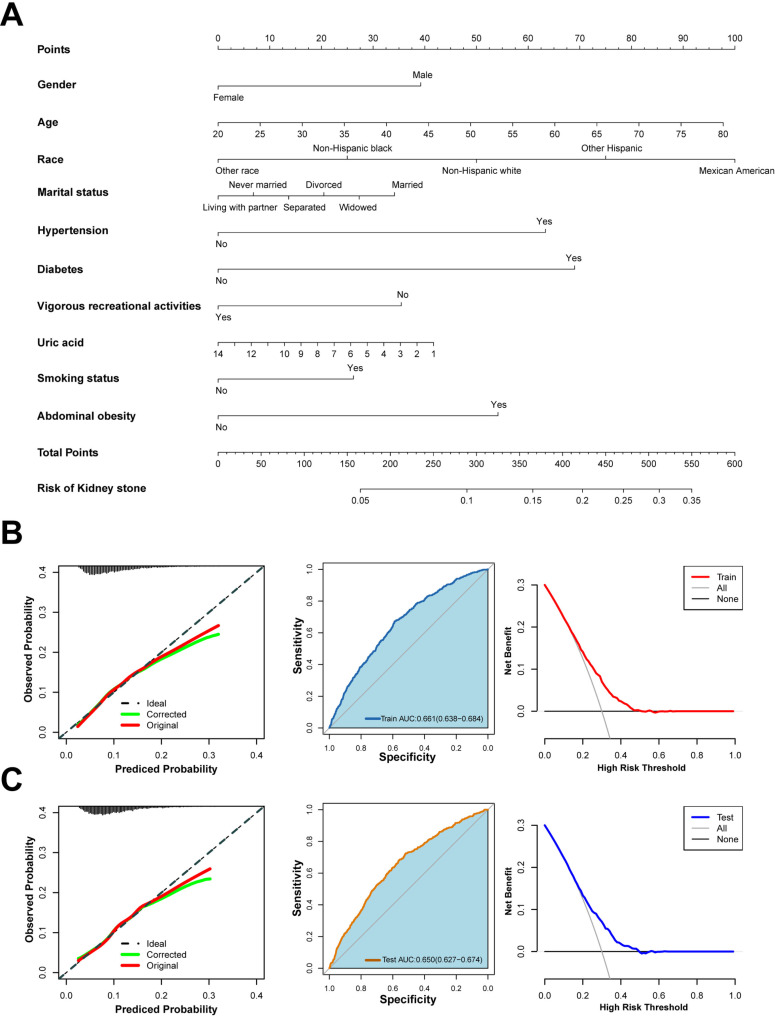
**A.** The nonogram of the predicted values of the KSD were calculated by scoring each numerical level for gender, age, race, marital status, hypertension, diabetes, vigorous recreational activity, uric acid, smoking status, and abdominal obesity. **B.** Calibration curve of the model constructed from the training set; ROC curve of the model constructed from the training set; DCA curve of the model constructed from the training set. **C.** Calibration curve of the model constructed from the test set; ROC curve of the model constructed from the test set; DCA curve of the model constructed from the test set.

A logistic regression model was developed using the results of the multivariate analysis. The dataset was divided equally, with 50% used for training and 50% for testing. A nomogram was constructed based on the training set to predict KSD prognosis (Fig 3A). Calibration curves demonstrated that the model’s predictions aligned closely with observed outcomes in both training and test sets, indicating strong calibration (Fig 3B and 3C).

ROC analysis yielded AUC values of 0.661 (95% CI: 0.638–0.684) for the training set and 0.650 (95% CI: 0.627–0.674) for the test set, confirming the model’s predictive accuracy (Fig 3B and 3C). To emphasize the superiority of SAD over traditional anthropometric measures, such as BMI and WC, this study compared previous findings, which reported AUC values below 0.6 for these measures [16,17], with the higher predictive performance of SAD (Fig 3B and 3C), DCA further validated the clinical utility of the nomogram in predicting KSD risk.

### Mediation effects modeling analysis

**Fig 4:**
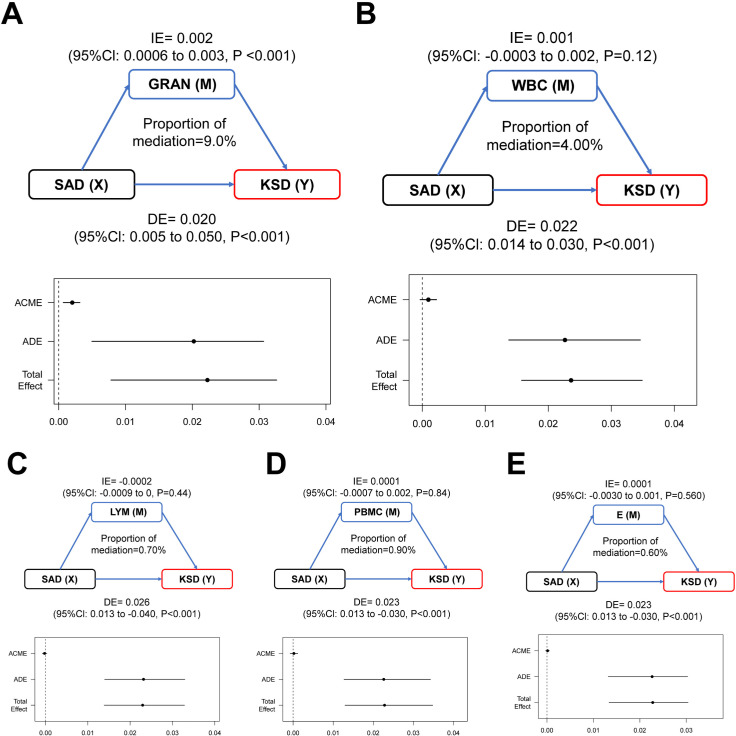
The diagram of intermediary effect. **A.** The mediation model shows the effects of SAD and GRAN on the KSD. **B.** The mediation model shows the effects of SAD and WBC on the KSD. **C.** The mediation model shows the effects of SAD and LYM on the KSD. **D.** The mediation model shows the effects of SAD and PBMC on the KSD. **E.** The mediation model shows the effects of SAD and E on the KSD. IE, indirect effect; DE, direct effect; Proportion of mediation =  IE/ (DE +  IE).

To explore the potential mechanism linking SAD and KSD, mediation analysis was conducted with neutrophils (GRAN), white blood cells (WBC), lymphocytes (LYM), monocytes (PBMC), and eosinophils (E) as mediating variables. The results (Fig 4) revealed that GRAN significantly mediated the relationship between SAD and KSD (P <  0.001). Mediation analysis decomposed the total effect (TE) of SAD on KSD into direct (DE) and indirect effects (IE), with the latter reflecting GRAN’s mediating role.

## Discussion

KSD is a prevalent condition, yet its etiology remains incompletely understood [18]. It is characterized by a high incidence and recurrence rate [19], making it a significant and urgent public health concern, particularly in economically underdeveloped regions, and a matter of global importance [20,21]. Understanding the risk factors and predictors of KSD is essential for its effective treatment and prevention.

We analyzed data from the NHANES database and found a positive association between SAD and the risk of KSD after adjusting for the covariates, including sex, age, race, marital status, hypertension, diabetes mellitus, vigorous recreational activity, uric acid, smoking, and abdominal obesity. Previous studies have highlighted the predictive value of SAD for conditions such as coronary artery disease, diabetes, and metabolic syndrome, but its role in predicting KSD remains unexplored [22–24]. Therefore, we propose using SAD as a predictive tool to achieve our research goals. SAD measures abdominal fat thickness and serves as an indicator of abdominal obesity [25]. Lee et al. demonstrated that obesity-related markers are associated with kidney stone prevalence and incidence in cross-sectional and longitudinal cohort studies. While BMI is commonly used to assess obesity-related risks, it primarily reflects overall weight relative to height and does not capture fat distribution, which may have a more direct role in the pathophysiology of KSD. In contrast, SAD provides a more specific measure of visceral fat accumulation, offering greater insight into the metabolic and physiological mechanisms underlying KSD. Kidney stones form when the balance between the solubility and precipitation of stone salts in the urinary tract and kidneys is disrupted. Obesity contributes to kidney stone formation through several mechanisms. One study found that excess body fat in KSD patients leads to uric acid supersaturation, thereby increasing the risk of uric acid stones compared to non-KSD individuals [26]. Calcium oxalate stones, which account for approximately 65% of all kidney stones, are influenced by obesity, as excessive body mass reduces the breakdown of oxalate and enhances its absorption by the intestinal flora. This dysregulation of uric acid and oxalate excretion is a known risk factor for calcium oxalate stones [26,27]. Furthermore, the accumulation of crystals and lipids in renal tubules can alter the microenvironment of kidney-associated cells, promoting the production of adipocytokines and macrophage formation, ultimately leading to kidney stone formation [28]. In lipid metabolism, fatty acid binding protein 4 (FABP4) plays a protective role against kidney stone formation, with knockdown of FABP4 accelerating stone development [29].

Our findings also suggests that SAD is not only a reliable physical measure with clinical utility for predicting KSD prognosis but may also influence KSD indirectly through GRAN. Neutrophils, which constitute 50–70% of all human leukocytes, are key inflammatory cells with phagocytic and migratory capabilities [30]. Although neutrophils are integral to immune responses, their role in kidney stone formation is poorly understood. Crystal-associated diseases, including kidney stones, silicosis, and atherosclerosis, are influenced by oxidative stress and inflammation [31–33]. In the case of KSD, oxalic acid crystals—the most common urinary crystals—induce oxidative stress and inflammatory responses, leading to the accumulation of inflammatory cells and the release of inflammatory mediators [34,35]. Notably, visceral fat accumulation, as indicated by SAD, promotes systemic inflammation, potentially driving GRAN expression. GRAN may then contribute to KSD development through its effects on inflammation and cellular processes that facilitate stone formation. Given the accessibility and cost-effectiveness of routine blood tests, incorporating these findings into KSD diagnosis and treatment warrants further exploration. Additionally, blood lipids, calcium, and phosphorus have been shown to influence KSD development and exhibit both direct correlations and mediating effects [14,15]. Future studies should integrate these parameters for a more comprehensive assessment.

This study has several strengths. First, we validated the correlation between SAD and KSD using a cross-sectional data from the large, nationally representative NHANES database, while adjusting for multiple correlates. Second, were the first to predict KSD risk using SAD by developing a predictive model and validating its accuracy and clinical relevance. Additionally, through mediational modeling, we established that SAD can influence KSD via GRAN. However, while our findings demonstrate an association between SAD and KSD, and a predictive model has been developed, SAD should be regarded as a risk factor rather than a diagnostic tool. Unlike imaging techniques such as X-rays or ultrasounds, which directly identify kidney stones, SAD provides an indirect measure of central obesity, which contributes to KSD risk. Consequently, SAD may be particularly useful in resource-limited settings or as part of broader screening initiatives to identify high-risk individuals.

Despite these strengths, the study has limitations. First, we were unable to conduct longitudinal analyses due to resource constraints, limiting the quality and depth of our conclusions. Further experimental studies are needed to confirm these associations. Second, as the findings are based on observational data from the NHANES database, they are subject to recall bias inherent in self-reported questionnaires, and we could not elucidate underlying biological mechanisms. Future research should integrate SAD with other biomarkers and clinical parameters, such as serum calcium and urinary oxalate, to improve predictive performance. Combining SAD with established risk factors and demographic characteristics could significantly enhance predictive accuracy and clinical utility. Advanced modeling techniques, including machine learning, could further refine these risk prediction models. Finally, external validation in independent cohorts is essential to ensure the robustness and generalizability of these findings.

## Conclusion

Our analysis of a cross-section of the U.S. population revealed that SAD is a stronger and more reliable predictor of KSD risk compared to traditional obesity indicators like BMI. The positive association between SAD and KSD risk remained significant even after adjusting for potential confounders, underscoring the robustness of this relationship. These findings establish SAD as a key risk factor for KSD, with the multivariate logistic regression model incorporating SAD demonstrating both clinical utility and predictive accuracy. Moreover, the analysis identified that SAD influences KSD through the mediating effect of the GRAN. Collectively, these results suggest that SAD has the potential to replace BMI as a more effective indicator for predicting KSD risk and informing clinical management strategies.
